# The role of tetrahydrocannabivarin (THCV) in metabolic disorders: A promising cannabinoid for diabetes and weight management

**DOI:** 10.3934/Neuroscience.2025003

**Published:** 2025-03-12

**Authors:** Scott Mendoza

**Affiliations:** Department of Biomedical Laboratory Science, Namseoul University, Cheonan 31020, Republic of Korea

**Keywords:** tetrahydrocannabivarin (THCV), metabolic disorders, obesity management, type 2 diabetes, cannabinoids, appetite suppression, glucose regulation, endocannabinoid system, insulin sensitivity

## Abstract

Disorders of the metabolism, including obesity and type 2 diabetes, represent significant global health challenges due to their rising prevalence and associated complications. Despite existing therapeutic strategies, including lifestyle interventions, pharmacological treatments, and surgical options, limitations such as poor adherence, side effects, and accessibility issues call attention to the need for novel solutions. Tetrahydrocannabivarin (THCV), a non-psychoactive cannabinoid derived from *Cannabis sativa*, has emerged as a promising agent to manage metabolic disorders. Unlike tetrahydrocannabinol (THC), THCV exhibits an antagonistic function on the CB1 receptor and a partial agonist function on the CB2 receptor, thus enabling appetite suppression, enhanced glucose regulation, and increased energy expenditure. Preclinical studies demonstrated that THCV improves insulin sensitivity, promotes glucose uptake, and restores insulin signaling in metabolic tissues. Additionally, THCV reduces lipid accumulation and improves the mitochondrial activity in adipocytes and hepatocytes, shown through both cell-based and animal research. Animal models further revealed THCV's potential to suppress appetite, prevent hepatosteatosis, and improve metabolic homeostasis. Preliminary human trials support these findings, thereby showing that THCV may modulate appetite and glycemic control, though larger-scale studies are necessary to confirm its clinical efficacy and safety. THCV's unique pharmacological profile positions it as a possible therapeutic candidate to address the multifaceted challenges of obesity and diabetes. Continued research should concentrate on optimizing formulations, undertaking well-designed clinical studies, and addressing regulatory hurdles to unlock its full potential.

## 1. Introduction

Type 2 diabetes and obesity are examples of metabolic diseases classified as modern-day epidemics, altering public health trajectories worldwide [1],[2]. Existing therapies for obesity and diabetes, including lifestyle interventions, pharmacological treatments, and bariatric surgery, offer varying levels of success [3]–[5]. However, limitations such as poor long-term adherence, adverse side effects, and accessibility challenges highlight the need for alternative approaches to effectively address these disorders [6],[7].

In recent years, the endocannabinoid system (ECS) has gained attention as a key controller of metabolic processes, appetite, and energy balance. Through CB1 and CB2 receptors, the ECS influences fat storage, glucose metabolism, and feeding behaviors, making it a promising target for metabolic interventions [8]. While synthetic CB1 antagonists can result in adverse psychiatric side effects, natural cannabinoids offer a safer and more effective alternative for the treatment of metabolic disorders [9]. Cannabis sativa derivatives possess anti-inflammatory, antioxidant, and neuroprotective properties that may help reverse metabolic issues [10]. Extensive research is currently underway in the United States to elucidate the benefits and risks of various cannabinoid compounds in both clinical and nonclinical contexts [11].

Tetrahydrocannabivarin (THCV), a lesser-known cannabinoid derived from *Cannabis sativa*, has emerged as a novel therapeutic candidate. Unlike tetrahydrocannabinol (THC), which stimulates appetite, THCV exhibits CB1 receptor antagonism and CB2 partial agonism, thus leading to appetite suppression, improved glucose regulation, and energy expenditure. These properties position THCV as a promising agent to maintain obesity and type 2 diabetes, thus offering an alternative pathway to address the limitations of current therapies [12]. A two phase, dose ranging, placebo-controlled trial that evaluated the safety and acute effects of THCV in healthy participants revealed that THCV exhibited a favorable safety profile, with most adverse events being mild; moreover, lower doses produced a preliminary signal for improved sustained attention, while higher doses resulted in mild THC-like effects [13].

This mini-review examines the current findings on the benefits of THCV in addressing obesity and diabetes challenges, thereby focusing on its pharmacological mechanisms, clinical applications, challenges, and future research directions.

## 2. Methods

A literature search was conducted using PubMed, Web of Science, and Google Scholar. The following keywords were used in various combinations: “tetrahydrocannabivarin,” “THCV,” “THC,” “CBD,” “obesity,” “type 2 diabetes,” “metabolic disorders,” “appetite suppression,” “cannabinoids,” “glucose regulation,” “endocannabinoid system,” “insulin sensitivity,” “risk factors,” “cannabinoid synthesis,” “pychosis,” “hyperemesis,” “regulatory issues,” and “Good Clinical Practice.” Additional articles were identified by screening the reference lists of relevant studies and reviews.

Studies were considered eligible if they (1) were published in English, (2) focused on the role of THCV in metabolic processes related to obesity or type 2 diabetes, and (3) reported original findings from preclinical or clinical research. Systematic reviews, meta-analyses, and narrative reviews that addressed THCV's metabolic effects were also included. Conference abstracts, case reports, non-English articles, and studies not directly relevant to THCV were excluded.

Key data extracted from each eligible publication included the study design, dosage, and administration of THCV, the primary outcomes (e.g., insulin sensitivity, body weight changes, glucose regulation, appetite metrics), and any reported adverse effects. The findings were qualitatively synthesized to provide an overview of THCV's potential efficacy and safety in managing obesity and type 2 diabetes, thus acknowledging the variability and limitations across studies.

## 3. Pharmacological profile of THCV

THCV exerts its effects by use of the endocannabinoid system (ECS), which is a series of molecules comprised of enzymes, receptors, and ligands that play a critical role in metabolic regulation. THCV has an antagonistic effect on the CB1 receptor and exhibits an agonist effect on the CB2 receptor, thus leading to its unique effects on appetite suppression, glucose metabolism, and inflammation [14]. An in vitro model demonstrated that THCV pre-treatment protects adipose-derived mesenchymal stem cells from endoplasmic reticulum stress by attenuating inflammatory responses and normalizing the unfolded protein response, which may underlie its beneficial effects on the overall metabolic homeostasis [15].

The central nervous system contains most of the CB1 receptors, though they can also be found in peripheral areas such as the liver and adipose tissue. Activation of CB1 receptors has been linked to increased appetite, energy storage, and insulin resistance [16]–[18]. THCV's antagonistic action on CB1 inhibits these effects, thus resulting in reduced food intake, glucose regulation, and the prevention of excessive energy storage [12],[14]. Notably, a placebo-controlled, double-blind crossover study demonstrated that an oral administration of 10 mg THCV over five days significantly reduced THC-induced increases in heart rates and cognitive impairment in healthy male volunteers, thereby reinforcing its antagonistic action on the CB1 receptor [19].

In contrast, the CB2 receptor is predominantly found in peripheral tissues, including the immune system, liver, pancreas, and adipose tissue. CB2 activation has demonstrated a reduction in inflammation and an improvement in insulin sensitivity, both of these being critical in the treatment of metabolic disorders [16]. THCV's partial agonism at CB2 receptors helps lower systemic inflammation and enhances glucose regulation [20],[21].

**Figure 1.**
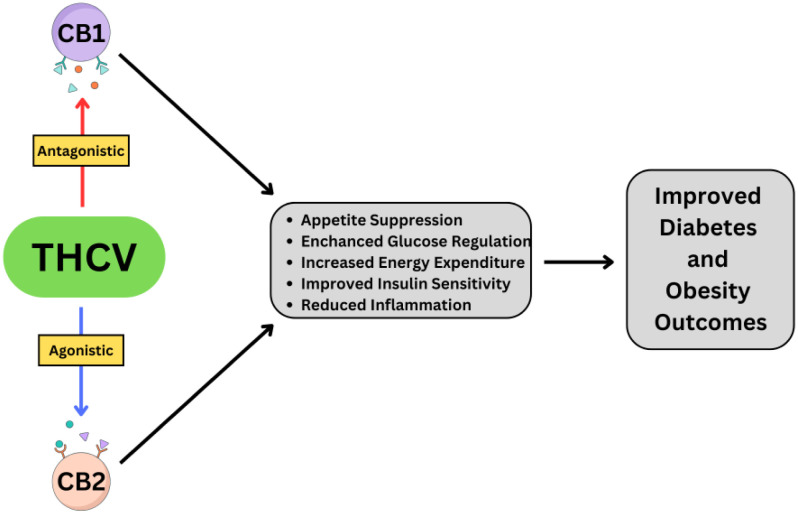
Mechanism of Action and Therapeutic Outcomes of THCV.

The dual mechanism of action of THCV is illustrated in Figure 1, thereby highlighting its role in appetite suppression, glucose regulation, and improved metabolic outcomes, which collectively support its therapeutic potential to manage obesity and type 2 diabetes.

## 4. THCV in diabetes management

### 4.1. Effects on insulin sensitivity and glucose homeostasis

Preclinical studies have demonstrated that THCV enhances insulin sensitivity, thus promoting glucose uptake in peripheral tissues. This process reduces insulin resistance, which is a hallmark of type 2 diabetes. By improving the efficiency of insulin action, THCV addresses one of the central mechanisms that contributes to hyperglycemia and disease progression [12],[14]. In dietary-induced obesity (DIO) mice, THCV increased the amount of energy used and reduced the glucose intolerance in a dose-dependent manner. In addition, THCV enhanced the tolerance to glucose and the sensitivity to insulin among genetically obese (ob/ob) mice, thereby restoring insulin signaling in hepatocytes and myotubes resistant to insulin [22]. Furthermore, studies have indicated that THCV's effects may involve signaling through GPR55, as shown in experiments that compared a GPR55 knockout and wild-type mice [23]. These findings suggest that GPR55-mediated mechanisms may partially account for THCV's ability to regulate glucose tolerance and energy balance, thus warranting a further investigation into its role in metabolic health.

THCV's dual modulation of CB1 and CB2 receptors allows it to effectively restore glucose homeostasis. By suppressing appetite-driven hyperglycemia through CB1 antagonism and improving insulin sensitivity via CB2 activation, THCV provides a comprehensive approach to glycemic control [12],[18].

### 4.2. Evidence from preclinical and human studies

While the majority of current evidence comes from preclinical studies, they provide strong support for THCV's potential in diabetes management. Animal models have consistently demonstrated improvements in insulin sensitivity, fasting glucose levels, and systemic inflammation [12],[22],[23]. Limited human trials have reported promising results in glycemic control and appetite regulation, paving the way for further research into its clinical applications. A trial with 62 diabetic human subjects showed promising results and demonstrated a lowered fasting plasma glucose and boosted pancreatic β-cell function [24].

The emerging evidence positions THCV as a novel therapeutic candidate for type 2 diabetes management. Its ability to address insulin resistance, lower fasting glucose levels, and restore glucose homeostasis makes it particularly relevant in a clinical setting. Going forward, studies should focus on randomized controlled trials on a larger scale to confirm the effects of THCV, establish the optimal dosing, and evaluate the long-term safety [8]. Additionally, studies that explore the combined effects of THCV with existing antidiabetic therapies could provide further insights into its potential role in comprehensive diabetes care.

## 5. THCV in appetite suppression and weight management

### 5.1. Appetite-suppressing properties compared to THC

THCV's appetite-suppressing effects are primarily mediated through its antagonistic action on the CB1 receptor. In contrast to THC, which activates CB1 receptors and stimulates appetite (commonly referred to as the “munchies”), THCV inhibits this receptor's activity [14]. This inhibition reduces food intake and prevents excessive caloric consumption, thus making it a promising agent for appetite regulation. Unlike THC, THCV's effects do not produce psychoactive side effects, thus further enhancing its therapeutic potential for weight management [14].

### 5.2. Evidence from animal studies

Animal studies have provided strong evidence for THCV's appetite-suppressing and anti-obesity effects. In some rodent models, THCV administration improved the energy expenditure by 30% over a 24-hour period [22]. Maintaining this level of energy expenditure over a longer period would limit weight gain. The experimental data demonstrated that THCV exhibits hypophagic properties, thus significantly reducing the food intake and weight gain in free-feeding mice at doses as low as 3 mg·kg^−1^. Interestingly, the hypophagic effects persisted without increased feeding on the following day [25]. THCV has demonstrated a possible role in weight management by directly reducing lipid accumulation in vitro, particularly among adipocytes and hepatosteatosis models. Through the use of nuclear magnetic resonance (NMR)-based metabolomics, the effects THCV caused in the metabolism of hepatocytes could be confirmed. These treatments induced post-translational modifications in the CREB, AMPKa2, PRAS40, and STATs proteins, suggesting an enhanced ability to metabolize lipids and the ability to cause an increase in the activity of cellular mitochondria, which are essential for energy expenditure. In vivo studies that used zebrafish and obese mice further demonstrated that THCV enhances yolk lipid utilization and prevents hepatosteatosis. These findings suggest that THCV may contribute to weight management by improving lipid metabolism and preventing fat accumulation, thus addressing key factors in obesity-related metabolic disorders [26]. These effects were observed without any significant adverse events, which supports its safety profile for further investigations as an alternative therapeutic treatment to obesity-related metabolic disorders.

### 5.3. Evidence from preliminary human trials

Preliminary human studies support the equivalent effects of THCV on appetite and weight regulation. In small-scale clinical trials, THCV has been shown to reduce hunger with patients that recorded a baseline numerical rating scale (NRS) score of 5.4 and a THCV treatment NRS score of 5.0. The NRS score evaluates appetite, with a 0 representing no appetite and a 10 representing the maximum appetite. However, it should be noted that this reduction in appetite was not found to be statistically significant [24]. Nonetheless, these effects are particularly promising for individuals with obesity or metabolic syndrome, where appetite dysregulation plays a significant role in the disease progression. In addition, a single 10 mg dose of THCV enhanced the neural responses to aversive food stimuli, such as moldy foods, thus suggesting a potential mechanism by which THCV may contribute to weight loss through the modulation of food reward and aversion [27]. Larger randomized controlled trials are needed and necessary to strengthen and confirm the significance of these findings and to establish the optimal dosing regimens.

## 6. THCV and metabolic disorders: A comparative overview

To highlight the unique advantages of THCV, it is essential to compare its mechanisms and therapeutic effects with other well-studied cannabinoids, including THC and cannabidiol (CBD). Table 1 provides a comparative overview.

**Table 1.** Comparison of THCV with other cannabinoids.

Cannabinoid	Mechanism of Action	Therapeutic Effects	Psychoactive Effects
THCV [12],[14],[22]–[24]	CB1 antagonist, CB2 partial agonist	Appetite suppression, improved glucose regulation, weight management	None
THC [28]–[30]	CB1 agonist	Appetite stimulation, analgesia	Psychoactive
CBD [28],[31]	Indirect CB1/CB2 modulation, anti-inflammatory	Anti-inflammatory, anxiolytic, neuroprotective	None

### 6.1. Reinforcing THCV's unique advantages

THCV stands out among cannabinoids due to its dual mechanism of action as an antagonist on CB1 and a partial agonist on CB2. Unlike THC, which promotes appetite and can lead to weight gain [29], THCV suppresses appetite and improves the energy balance [14]. Furthermore, THCV's ability to enhance glucose regulation makes it particularly relevant to manage both obesity and type 2 diabetes [22]. Compared to CBD, which primarily exerts anti-inflammatory and neuroprotective effects [31], THCV's targeted metabolic benefits offer a distinct therapeutic advantage for individuals with metabolic disorders [12].

These properties collectively position THCV as a unique and promising candidate to address the multifactorial challenges associated with obesity and type 2 diabetes. Further research will need to fully validate its clinical efficacy and explore its integration with existing treatment strategies.

## 7. Challenges and future directions

### 7.1. Limitations in clinical trials and evidence

Despite promising preclinical and limited human studies, the clinical evidence for THCV remains insufficient. Existing human trials are small in scale and often lack the statistical power needed to draw definitive conclusions [24]. Larger, randomized controlled trials (RCTs) with diverse populations are essential to validate THCV's efficacy, determine the optimal dosing, and assess its long-term benefits in managing obesity and type 2 diabetes. Recent advances in gene editing and fermentation technology now allow for the biosynthesis of cannabinoids in heterologous systems such as yeast and microalgae, thus possibly facilitating more meaningful clinical research by providing a cost-effective and reliable source of cannabinoids such as THCV [32],[33].

### 7.2. Formulation and bioavailability issues

One of the significant challenges in cannabinoid-based therapies, including THCV, is poor bioavailability. THCV has a low water solubility, which limits its absorption and systemic availability when orally administered [34]. To address this, innovative drug delivery platforms such as nanoformulations, lipid-based carriers, and emulsified preparations need to be developed to improve THCV's pharmacokinetics and therapeutic efficacy.

### 7.3. Safety and long-term studies

The long-term safety of THCV remains unclear due to the lack of extended clinical studies. While preclinical studies have shown no significant adverse effects [22],[24], the safety profile of chronic THCV administration in humans must be rigorously evaluated. Additionally, its potential interactions with other metabolic or weight-loss therapies warrant careful investigations to ensure a safe co-administration in clinical settings.

### 7.4. Addressing regulatory barriers

Cannabinoid-based therapies face significant regulatory hurdles that can delay clinical research and approval processes [35]. Standardized guidelines for THCV's production, dosing, and safety testing are required to accelerate its clinical translation and integration into therapeutic protocols. Additionally, regulatory frameworks must address the public concerns surrounding cannabinoids while ensuring that high-quality, evidence-based treatments are accessible to patients.

### 7.5. Future research directions

Therefore, future research efforts should focus on the following:

- Conducting large-scale, multicenter RCTs to establish THCV's efficacy and safety;
- Developing innovative formulations to enhance THCV's bioavailability and therapeutic potential;
- Investigating the long-term effects of THCV on metabolic health;
- Exploring THCV's synergistic effects when combined with existing antidiabetic or anti-obesity medications; and
- Addressing the regulatory landscape to facilitate the clinical adoption of cannabinoid-based therapies.

By addressing these challenges, THCV has the potential to become a widely accepted therapeutic option to manage obesity and type 2 diabetes, thereby offering targeted benefits that complement existing treatment strategies.

## 8. Conclusions

THCV appears to modulate key metabolic pathways including appetite regulation [17], glucose homeostasis [18], and insulin sensitivity [22] by antagonizing CB1 receptors and partially activating CB2 receptors [12],[14]. Early data from preclinical models and limited human trials suggest potential benefits for conditions such as obesity and type 2 diabetes.

Although the current evidence indicates that THCV holds promise to aid in the treatment of diabetes and obesity, the current data do not support its use as a therapeutic agent at this time. Rigorous basic and clinical studies conducted in compliance with FDA guidelines such as Good Manufacturing Practice, Good Laboratory Practice, and Good Clinical Trials Practice are needed before THCV can be approved for clinical use [36]. Additionally, it is important to note that the current research cautions against the use of unapproved cannabis products, which may only contain minimal amounts of THCV alongside containing high levels of THC, as these have been associated with serious adverse health outcomes such as psychosis and cannabis hyperemesis syndrome [37],[38].

Metabolic disorders are multifaceted and influenced by a range of genetic, environmental, and lifestyle factors, which makes the use of THCV as a single “one size fits all” intervention unlikely [39]. Nonetheless, THCV represents a promising, yet still exploratory, adjunct candidate in the management of metabolic disorders, thus warranting further rigorous investigation to fully establish its therapeutic role.

## Use of AI tools declaration

The authors declare they have not used Artificial Intelligence (AI) tools in the creation of this article.
